# Compound salvia pellet might be more effective and safer for chronic stable angina pectoris compared with nitrates: A systematic review and meta-analysis of randomized controlled trials

**DOI:** 10.1097/MD.0000000000014638

**Published:** 2019-03-01

**Authors:** Wei Huiping, Wang Yu, Jin Pei, Li Jiao, Zhang Shian, Jiang Hugang, Wang Zheng, Li Yingdong

**Affiliations:** a Affiliated Hospital of Gansu University of Chinese Medicine; b Gansu University of Chinese Medicine; c Gansu Institute of Integrated Chinese and Western medicine; d Key Laboratory of Traditional Chinese Medicine for Prevention and Treatment of Chronic Diseases; e School of Basic Medicine of Lanzhou University, Lanzhou, Gansu, China.

**Keywords:** chronic stable angina, compound salvia pellet, meta-analysis, nitrates

## Abstract

**Background:**

Chronic stable angina (CSA) resulted in a considerable burden for both individuals and the society. In this study we aimed to critically evaluate the effectiveness and safety of Compound salvia pellet compared with nitrates in the treatment of Chronic Stable Angina (CSA) pectoris, and to provide more credible evidence for clinical practice.

**Methods:**

A comprehensive and exhaustive search strategy was formulated to identify potential RCTs of compound salvia pellet for CSA in international and Chinese databases from their inception to July 4th, 2018. We also searched the bibliographies of relevant studies. Two reviewers independently assessed the quality of included trials by using Cochrane Risk of Bias Tool.

**Results:**

The literature search yielded 1849 citations and 51 RCTs (n = 4732) were included for meta-analysis after titles, abstracts and full text selection according to eligibility criteria. The pooled results suggested that compound salvia pellet was much more effective than nitrates in the improvement of angina symptoms (therapy = 4 weeks, RR = 1.23, 95%CI = [1.17, 1.30], *P* < .001, *I*^2^ = 0%; therapy = 4 weeks, RR = 1.13, 95%CI = [1.08, 1.17], *P* < .001, *I*^2^ = 45.6%), and ECG test (therapy = 4 weeks, RR = 1.24, 95%CI [1.14, 1.35], *P* < .001, *I*^2^ = 51.5%; and therapy > 4 weeks, RR = 1.30, 95%CI[1.20, 1.42], *P* < .001, *I*^2^ = 36.4%) in CSA. Compared with nitrates, the percentage of patients with adverse events significantly decreased when prescribed with compound salvia pellet (3.2% vs 17.0%).

**Conclusion:**

Compound salvia pellet might be more effective on the improvement of angina symptoms, ECG test and with few adverse events compared with nitrates. While there are some limitations in this study, which may weaken the results, we believe the findings could provide useful information for stakeholders concerned with outcomes in patients with CSA. More rigorous RCTs with high quality are needed to confirm these findings.

## Abstract 2

Supplemental Digital Content is available in the text

## 1 Introduction

Chronic stable angina (CSA) is a pain or constricting discomfort that typically occurs in the front of the chest and is brought on by physical exertion or emotional stress,^[1]^ which affects over 9 million adults in the United States, with an estimate of 500,000 new cases annually.^[2]^ CSA resulted in a considerable burden for both individuals and the society. At present, organic nitrates are indicated as the first-line therapy for the long-term management of CSA.^[3–6]^ It has been demonstrated that nitrate treatment provides control of angina symptoms and improves the quality of life.^[7]^ But a major limitation of using nitrates is the development of tolerance, defined as the loss of hemodynamic and antianginal effects during sustained therapy.^[8,9]^

Traditional Chinese Medicine (TCM), Radix salviae miltiorrhizae (Danshen) and its compound formula as drugs (compound salvia pellet) provides an alternative option for CSA. Experimental studies have shown that compound salvia pellet can dilate coronary arteries, increase coronary blood flow, and scavenge free radicals^[10]^ in ischemic diseases, which could promote blood circulation to activate blood and remove blood stasis. Compound salvia pellet consist of active herbal ingredients extracted from Danshen (salviae miltiorrhizae), Sanqi (panax notoginseng), and Borneol (Cinnamomum camphora).^[11]^ Compound salvia pellet had been recommended by CSA guidelines in China,^[12]^ and had been selected as an essential drug for CSA by Chinese government since 2009. That is to say, the cost of compound salvia pellet could be covered by medical insurance in China.

Although compound salvia pellet had been widely used in China, no strong evidence was found to support the recommendation and policy decision. Until now, many randomized controlled trials (RCTs) had been published, which indicated that compared with the organic nitrates, the compound salvia pellet might be effective in patients with CSA. Therefore, considering these backgrounds, the aim of this study is to critically evaluate the effectiveness and safety of compound salvia pellet for CSA, hoping to provide more credible evidence for clinical practice.

## 2 Methods

### 2.1 Searched databases and search strategies

A comprehensive and exhaustive search strategy was formulated to identify all relevant studies regardless of language or publication status (published, unpublished, in press, and in progress). Pubmed, Embase, Cochrane Controlled Trials register were searched as international databases and Wanfang Data Knowledge Service Platform, VIP Online Publishing Platform, China National Knowledge Infrastructure (CNKI) and SinoMed were searched as domestic databases to identify RCTs of compound salvia pellet for CSA. The search terms consisted “compound salvia pellet”, “Danshen pill”, “Danshen droplet pill” and “angina”. Database searches were initially conducted at October 31st, 2016, and updated at July 4th, 2018, detailed searching information could be found in Appendix 1. Furthermore, a manual search was performed of the bibliographies of studies and reviews. Some trials reported data that were insufficient for statistical pooling, and then corresponding authors were contacted to provide additional data.

### 2.2 Including criteria

The articles were reviewed by 2 reviewers and studies were selected systematically according to predefined criteria. Studies were required to meet the following criteria:

1. Type of studies: Randomized controlled trials (RCTs) will be considered for inclusion regardless of publication status and language of publication. Trials with quasi-random designs will not be considered for inclusion.
2. Type of patients: adults with a diagnosis of CSA more than 1 month. Appropriate participants will be included regardless of gender, race, and educational status of CSA.
3. Type of interventions: Trials that compared compound salvia pellet for treatment of patients with CSA compared with nitrates were considered for inclusion, regardless of therapy time and follow up duration.
4. Type of outcome measures: the improvement of angina symptoms (angina times after intervention in 1 week happens half or less than treatment before) and reduced angina attack were defined as primary outcome.

The improvement of electrocardiogram (ECG) (ST segment of ECG recovery to be normal), and adverse events during the treatment were defined as secondary outcome.

### 2.3 Data extraction and quality assessment of trials

Data were extracted independently by 2 authors using a standard form. Data extracted include:

1. general information (e.g., title, authors, reference, language, year of publication, and setting);
2. trial characteristics related to methodological quality (e.g., design, duration of follow up, sequence generation, allocation sequence concealment, and blinding);
3. intervention and comparison (dose, route, and timing);
4. patients information (e.g., baseline characteristics and diagnostic criteria);
5. outcomes data (e.g., events, estimates, standard error, and *P* value).

Discrepancies were resolved by discussion. Data on the number of patients with each outcome event by allocated treatment group, irrespective of compliance or follow-up, were sought to allow an intention-to-treat analysis. If the above data were not available in the trial reports, further information was sought by correspondence with the principal investigator.

The methodological quality of included randomized trials was assessed and reported according to Cochrane Collaboration's handbook to assess the risk of bias.^[13]^ The methodological quality assessed included:

1. sequence generation,
2. allocation sequence concealment,
3. blinding of participants and personnel,
4. blinding of outcome assessment,
5. incomplete outcome data,
6. selective reporting and
7. other potential sources of bias.

The evaluation of methodological quality was performed independently by the 2 reviewers, and discrepancies were solved through discussion.

### 2.4 Sensitivity analysis

Sensitivity analyses were performed to check the influence of the removed data set to the overall estimates effects through deleting one single study from the overall pooled analysis each time.

### 2.5 Assessment of publication bias

We used Egg test^[14]^ to assess publication bias and other small-study effects in a qualitative manner for comparisons and outcomes in which more than 7 trials were included.

### 2.6 Summary of findings

We used the principles of GRADE (Grades of Recommendation, Assessment, Development, and Evaluation) system^[15]^ in our review to assess the quality of the body of evidence associated with specific outcomes and constructed a 'Summary of findings’ (SoF) table. The GRADE approach is used to assess the quality of a body of evidence based on the extent to which one can be confident that an estimate of effect or association reflects the item being assessed. Assessment of the quality of a body of evidence considers study methodological quality, directness of the evidence, heterogeneity of the data, precision of the effect estimates and risk of publication bias.

### 2.7 Data analysis

We pooled data using Stata statistical software version 12.0 (Stata Corp, College Station, TX) and implemented the meta-analysis with the random effect model for the potential heterogeneity between the trials. Relative Risk (RR) was used to construct forest plots of binary variable data, standard mean difference (SMD) with 95% confidence intervals (CI) was used to construct forest plots of continuous data. *P* < .05 was considered statistically significant. The presence of heterogeneity across the studies was evaluated using the Q statistic quantified with the *I*^2^ statistic. The heterogeneity was considered as significant when *P* value was not bigger than .10. Heterogeneity could be quantified by *I*^2^ statistic with 75%, 50%, and 25% that, respectively, corresponds to high, moderate, and low heterogeneity.^[16]^

## 3 Results

### 3.1 Search results and study characteristics

**Figure 1:**
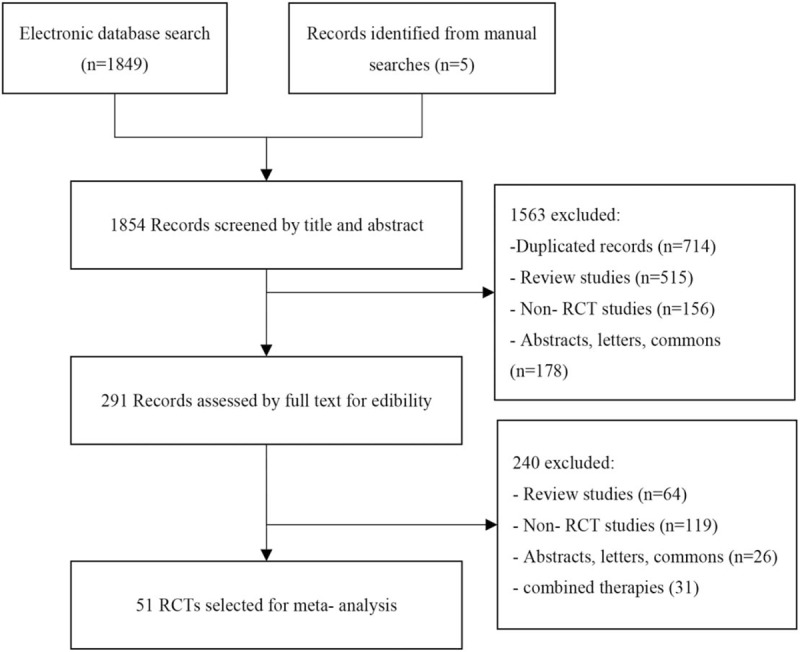
The flow chart of systematic studies search and selection procedure.

The literature search yielded 1849 citations from electronic database concerning compound salvia pellet for CSA (Fig. 1). After the 2 reviewers screened the titles, abstracts and full text according to inclusion criteria, 1563 articles were excluded as duplicates, non-clinical studies, obvious error, or study objectives different from the aim of this review. Finally, 51 RCTs^[17–67]^ with 4732 patients (2511 received compound salvia pellet and 2221 received nitrates) were identified, none of them was multi-center study. Forty-six studies were given the dose of compound salvia pellet of 250 mg, tid, 4 were 250 mg, tid and the last 2 were 200 mg, tid and 1 was 125 mg, tid respectively (Table 1). Patients in control group received isosorbid dinitrate ranged from 8 to 20 mg, tid. The course of disease in most included studies over 1 year and the therapy period in 18 studies was 4 weeks, 6 weeks in 4 studies, 8 weeks in 24 studies, 10 weeks in 1 studies and 12 weeks in 3 studies. More characteristics information about included RCTs could be seen in Table 1.

**Table 1:**
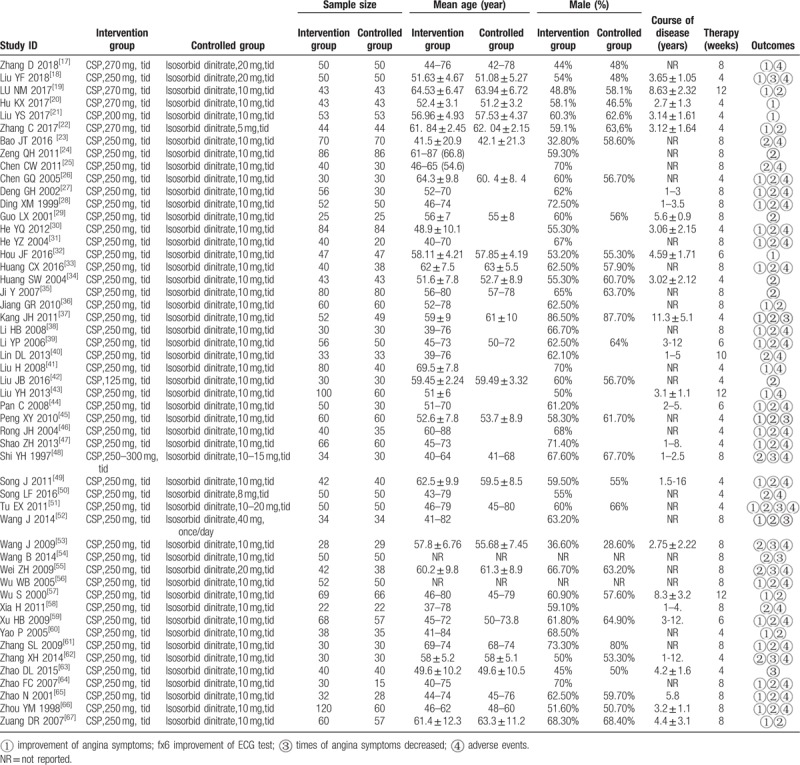
Characteristics of included studies.

### 3.2 Methodological quality of the included studies

**Figure 2:**
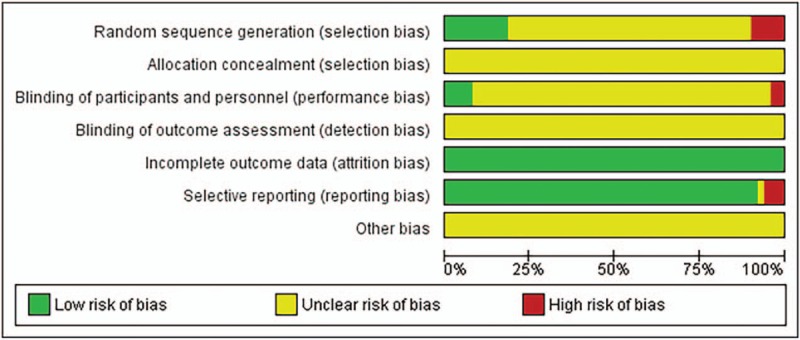
Risk of bias graph about each risk of bias item presented as percentages across all included studies.

**Figure 3:**
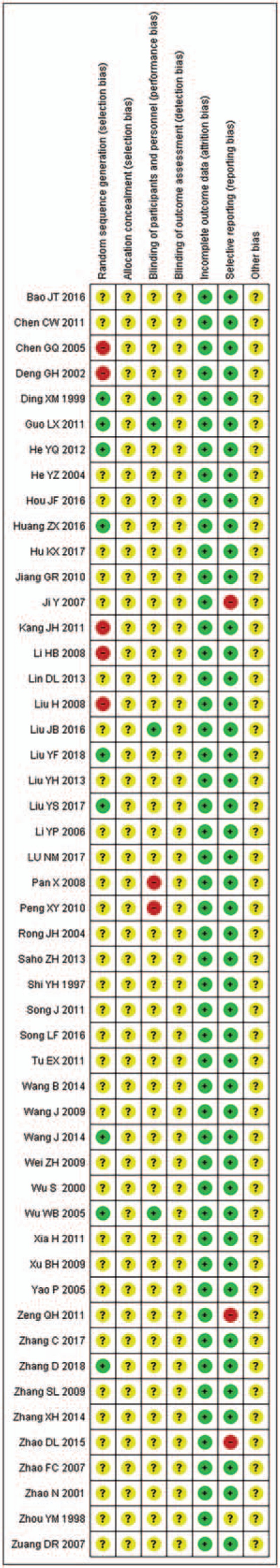
Risk of bias summary about each risk of bias item for each included study.

Nine studies^[17,18,21,28–30,33,52,56]^ with low risk in random sequence generation, 4 studies^[28,29,42,56]^ with low risk in blinding of participants and personnel, all studies with low risk in incomplete outcome data, and 47 studies with low risk in selective reporting, and none studies with low risk in allocation concealment and blinding of outcome assessment. (Figs. 2 and 3). No trials described a beforehand sample size calculation. The average sample size of the included trials was 95 patients, varying from 44 to 180, and there were more than 100 subjects in 23 trials (45.1%). The homogeneity test for the difference between the treatment group and the control group with a *P* value was used in 16 trials, the other trials only described the baseline levels without statistical analysis, and therefore it was difficult to make sure whether the 2 groups were comparable in all aspects.

### 3.3 Compound salvia pellet versus nitrates

**Figure 4:**
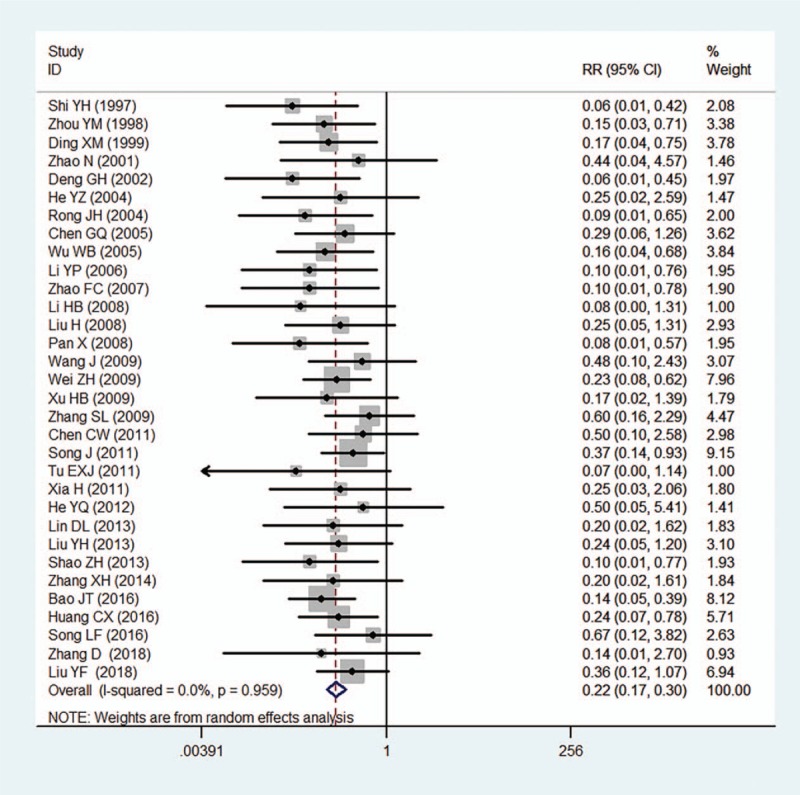
A meta-analysis of improvement of angina symptoms for compound salvia pellet versus nitrates.

Forty-four studies (n = 4149) reported improvement of angina symptoms. Pooled results showed compound salvia pellet had significant effect on the improvement of angina symptoms compared with nitrates (RR = 1.16, 95%CI = [1.12, 1.20], *P* < .001), with moderate heterogeneity found in the pooled estimates (*I*^2^ = 46.3%). Stratified analysis was conducted in terms of treatment durations. Pooling data showed a significant difference was found in the improvement of angina symptoms of compound salvia pellet group with a 4-week treatment duration (RR = 1.23, 95%CI = [1.17, 1.30], *P* < .001, *I*^2^ = 0%) and over 4-week treatment duration (RR = 1.13, 95%CI = [1.08, 1.17], *P* < .001, *I*^2^ = 45.6%) (Fig. 4).

**Figure 5:**
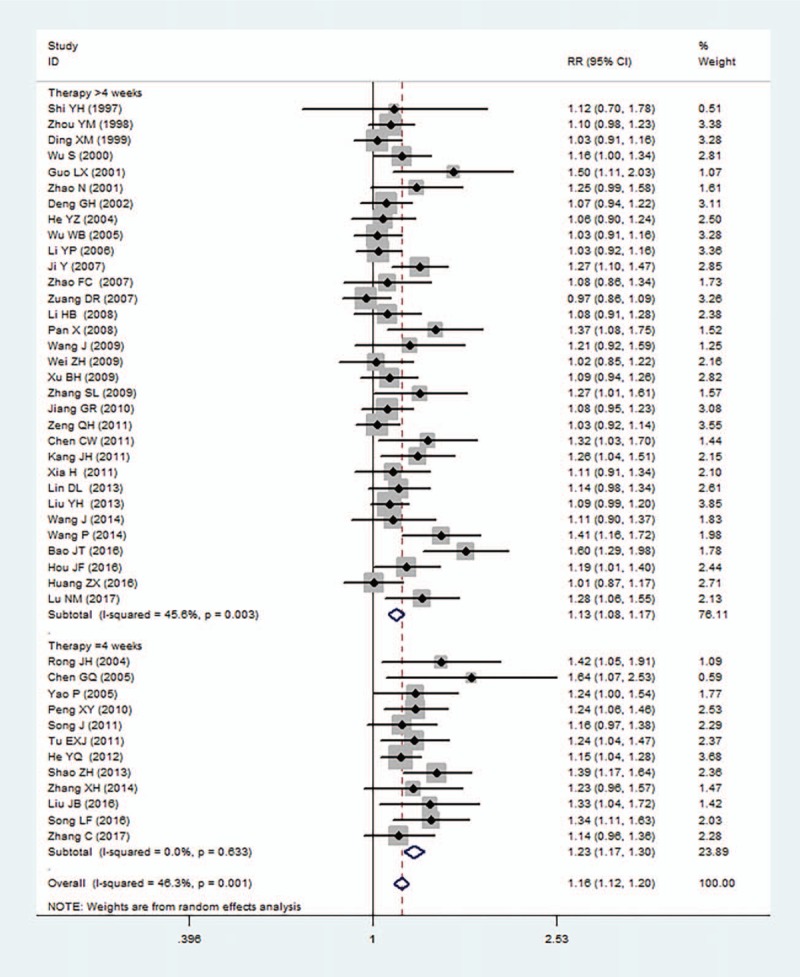
A meta-analysis of improvement effect of ECG for compound salvia pellet versus nitrates. ECG = electrocardiogram.

The ECG test was used in 35 trials (n = 3419). The meta-analysis of the ECG test suggested that treatment with compound salvia pellet had a significant improvement effect of ECG compared with nitrates (RR = 1.28, 95%CI = [1.20, 1.36], *P* < .001), low heterogeneity was found in the pooled estimates (*I*^2^ = 45.4%). Furthermore, compound salvia pellet showed greater increased effect on the improvement of ECG with both 4 weeks and over 4 weeks of treatment durations (RR = 1.24, 95%CI [1.14, 1.35], *P* < .001, *I*^2^ = 51.5%; RR = 1.30, 95%CI [1.20, 1.42], *P* < .001, *I*^2^ = 36.4%, respectively) (Fig. 5).

**Figure 6:**
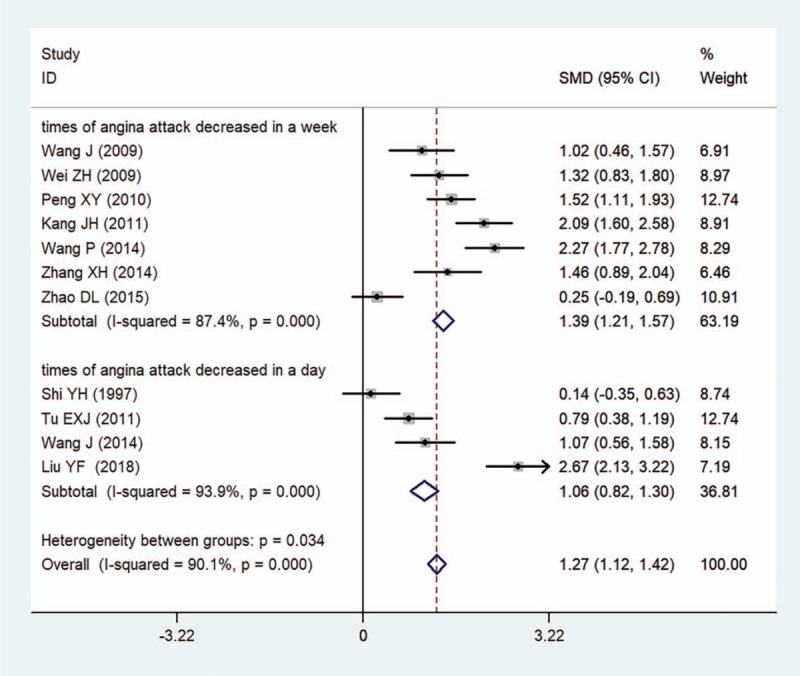
A meta-analysis of times of angina symptoms decreased for compound salvia pellet versus nitrates.

Times of angina symptoms decreased was reported in 11 studies (n = 930). The pooled results indicated that compound salvia pellet group had less times of angina attack decreased within a week and a day (SMD = 1.39, 95%CI = [1.21, 1.57], *I*^2^ = 87.4%; SMD = 1.06, 95%CI = [0.82, 1.30], *I*^2^ = 93.9%, respectively) (Fig. 6).

**Figure 7:**
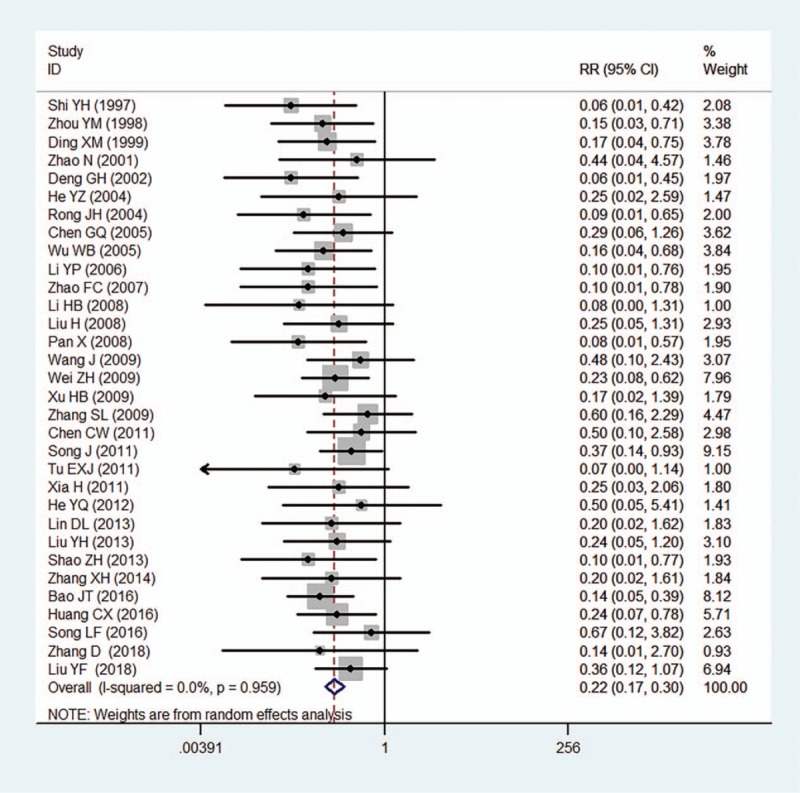
A meta-analysis of adverse events for compound salvia pellet versus nitrates.

Adverse events were reported in 55 of 1723 (3.2%) patients treated with compound salvia pellet, among which, abdominal complaints, nausea, and dyspepsia were mostly reported, and no dropouts or withdrawals for adverse events was founded in compound salvia pellet group. In patients treated with nitrates, adverse events were reported in 248 of 1461 subjects (17.0%), and there were 21 withdrawals for some serious complaints such as flush, dizziness, headache, and syncope. The pooled results indicated the rate of adverse events in compound salvia pellet group was significant lower than nitrates group (RR = 0.22, 95%CI = [0.17, 0.30], *P* < .001), no heterogeneity was found in the pooled estimates (*I*^2^ = 0%) (Fig. 7).

### 3.4 Sensitivity analysis

We deleted 1 single study from the overall pooled analysis each time to check the influence of removed data set to the overall RRs. Finally, the sensitivity analysis showed that the pooled results were stable, which improved our confidence to compound salvia pellet for CSA.

### 3.5 Publication bias

Publication bias assessment of each outcomes by Egg test were presented in Table 2. Weighted linear regression of effect estimates on their standard error revealed evidence of publication bias or other small-study effects in outcomes of improvement of angina symptoms (therapy > 4weeks and therapy = 4 weeks) and improvement of ECG (therapy = 4 weeks).

**Table 2:**
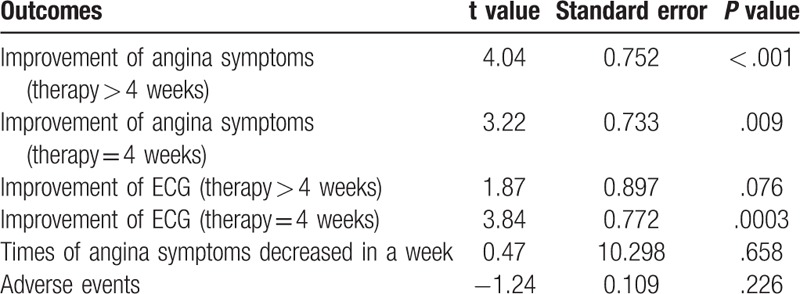
Publication bias assessment by Egg test.

## 4 Discussion

This meta-analysis of randomized trials suggested that compound salvia pellet was more effective than nitrates in the improvement of angina symptom, ECG test and times of angina symptoms decreased. After stratified analysis was conducted in terms of treatment durations, pooled results showed significant differences in the improvement of angina symptoms (Fig. 4) and ECG test (Fig. 5) in compound salvia pellet group with both 4-week treatment duration and over 4-week treatment duration. The advantage of compound salvia pellet was still obvious when subgroup analysis was conducted according to the times of angina symptoms decreased counted in a day or a week (Fig. 6). In addition, compound salvia pellet seemed to be safer than nitrates, as compound salvia pellet had lower adverse events rate compared with nitrates (3.2% vs 17.0%).

These main findings of the review are presented in summary of findings for the main comparison (Table 3). However, none of the included trials, reported endpoint events with long-term follow-up duration, such as mortality, and quality of life, which actually are more useful for evaluating the effectiveness of the 2 arms.

**Table 3:**
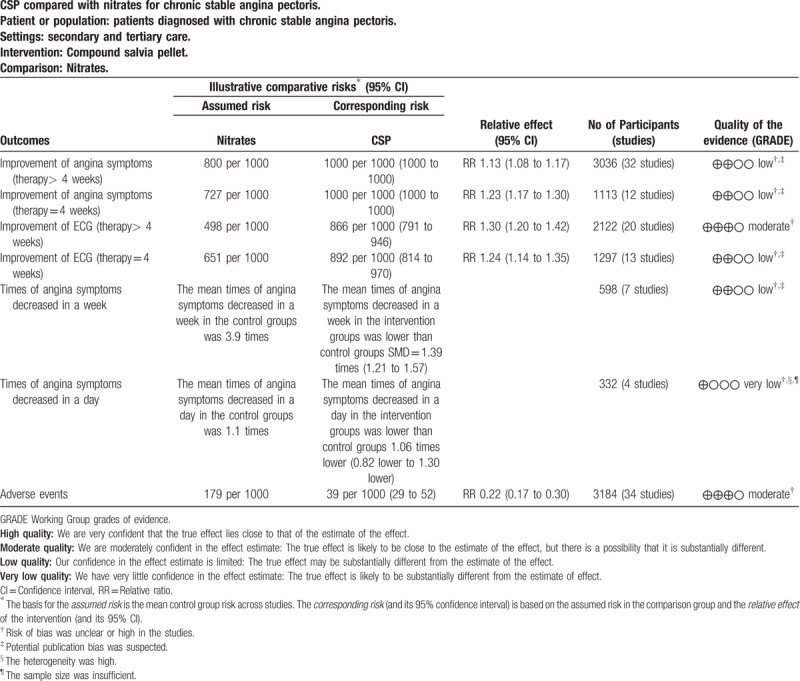
Summary of findings for the main outcomes.

Patients with angina are at substantially higher risk of cardiac death or myocardial infarction than general population. Therapies for patients with chronic stable angina aims at reducing symptoms, so as to reduce the risk of death and myocardial infarction. Symptomatic therapy is targeted at either reducing oxygen demand by decreasing the work of the heart or increasing oxygen supply by dilating coronary arteries.

The reason why compound salvia pellet showed a superior effect than nitrates perhaps due to their different active ingredients and pharmacological mechanism. Compound salvia pellet protects cardiomyocytes against myocardial ischemia and inhibits apoptosis via the Akt-eNOS signaling pathway.^[68]^ Moreover, it may have extensive effects on four metabolites (hypoxanthine, xanthine, inosine, and allantoin) in the pathway of purine metabolism which contribute to a decrease of oxygen-free radical.^[69]^ While nitrates dilate veins, arteries, and coronary arteries by relaxing vascular smooth muscle. They produce these effects by entering vascular smooth muscle cells where they are metabolized to 1, 2-glyceryl dinitrate and nitrite, via mitochondrial aldehyde dehydrogenase-2 (ALDH2 or mtALDH), and then nitric oxide and S-nitrosothiols.^[70]^ Sulfhydryl groups on ALDH2 are required for activity.

This review examined data from 51 studies with 4732 participants diagnosed with CSA, which formed the basis of this review certainly provided a considerable body of evidence. The overall methodological quality of the included studies was low, with most studies assessed as having one or more domains of unclear or high risk of bias. In light of the subjective nature of most of the outcomes of this review, the greatest concern was lack of adequate blinding which might result in big bias in outcomes measured. The quality of evidence of outcomes in this review were presented in Table 3, the quality of evidence of improvement of ECG (therapy > 4 weeks) and adverse events were rated to be “moderate” level, which indicated that the true effect was likely to be close to the estimate of the effect. While the quality of evidence of the other outcomes were rated to be “low” or “very low”, which suggested that our confidence in the effect estimate is limited.

Heterogeneity was moderate in the assessment of improvement in both symptoms and ECG test. Possible reasons for the heterogeneity are:

1. misuse of randomization,
2. different criteria for assessing the therapeutic efficacy, and
3. the kinds of nitrates with different quality criteria from different pharmaceutical companies. And heterogeneity was statistically significant in the outcome of times of angina symptoms decreased.

One of the main reasons might be, for several studies, the central tendency of the data was reported as a median rather than as a mean, and the spread was reported as a range or interquartile range. These data were approximated to mean and standard deviation by using the techniques described in Hozo study.^[71]^ However, it is important to note that these approximations may differ from the reported mean and standard deviation statistics. Another possible reason that contributed to the high heterogeneity might be misuse of randomization.^[72–74]^

It was significant that most important information in the included trials did not report adequately, which led to unclear risk of bias in most domains. Investigators and editors have developed the CONSORT (Consolidated Standards of Reporting Trials) statement to help authors improve reporting quality of RTCs by referring to the checklist and flow diagram.^[75]^

Although several previous meta-analyses had been published,^[76–79]^ the results from this systematic review are more reliable because

1. their studies mixed stable and unstable angina pectoris together, which could confuse potential readers,
2. their studies provided no subgroup analysis according to therapy duration or some other factors, which is a significant bias to heterogeneity,
3. their conduct did not follow PRISMA requirements, and
4. their results had been outdated because of new trials published in recent years.

By contrast, this systematic review includes only the RCTs comparing compound salvia pellet and nitrates. The interpretation of the pooled results is used by GRADE approach, and subgroup and sensitivity analyses were conducted to avoid possible biases of specific groups of studies.

This review also has some limitations:

1. most of the included trials were of low metrological quality, with high or unclear risk of bias random sequence generation, allocation concealment and blinding;
2. the course of disease ranged from 1 to 16 years, and the diagnostic criteria to CSA varied because the studies published from 1997 to 2018 which might result in heterogeneity;
3. no trials described the beforehand sample size calculation and the allocation concealment.

## 5 Conclusions

This meta-analysis suggests that compound salvia pellet might be more effective on the improvement of angina symptoms, ECG test and with few adverse events compared with nitrates. Therefore, compound salvia pellet might be used as an alternative option for nitrates in the treatment of CSA. While there are some limitations in this study, which may weaken the results, we believe the findings could provide useful information for stakeholders concerned with outcomes in patients with CSA. More rigorous RCTs with high quality are needed to confirm these findings.

## Acknowledgments

The authors thank Janne Estill (Institute of Social and Preventive Medicine, University of Bern, Switzerland) and Editage Company for providing assistance with editing the language of final article.

## Author contributions

W.H.P, W.X.Q, W.Y, and Y.L had full access to all of the data in the study and take responsibility for the integrity of the data and the accuracy of the data analysis. W.H.P and W.X.Q contributed to drafting and critical revision of the article; W.H.P, J.P, and W.Z contributed to the data extraction, and data analysis; Y.K.H and L.Y.D. contributed to the study design and critical review. All authors contributed to the interpretation of study data and critically reviewed and approved the article before submission.

**Conceptualization:** Wei Huiping, Wang Yu, Li yingdong.

**Data curation:** Wei Huiping, Wang Yu, Li yingdong.

**Formal analysis:** Wei Huiping, Wang Yu, Li Jiao, Jiang Hugang, Li yingdong.

**Funding acquisition:** Wei Huiping, Wang Yu.

**Investigation:** Wei Huiping, Wang Yu.

**Methodology:** Wei Huiping, Jin Pei, Jiang Hugang, Wang Zheng, Li yingdong.

**Project administration:** Wang Zheng.

**Resources:** Wei Huiping, Jin Pei, Li Jiao, Zhang Shian, Wang Zheng.

**Software:** Wei Huiping, Jin Pei, Wang Zheng.

**Supervision:** Wei Huiping, Jin Pei, Wang Zheng, Li yingdong.

**Validation:** Wei Huiping, Jin Pei, Zhang Shian, Wang Zheng.

**Visualization:** Wei Huiping, Wang Zheng.

**Writing – original draft:** Wei Huiping.

**Writing – review & editing:** Wei Huiping, Li yingdong.

## Supplementary Material

Supplemental Digital Content
