# Supplementary material for: Compound salvia pellet might be more effective and safer for chronic stable angina pectoris compared with nitrates: A systematic review and meta-analysis of randomized controlled trials
Source: Medicine (Baltimore). 2019 Mar 1;98(9):e14638. doi: 10.1097/MD.0000000000014638 (PMC6831215; doi:10.1097/MD.0000000000014638)
Supplement: Supplemental Digital Content [file medi-98-e14638-s001.docx]

Appendix 1. Database search strategies

PUBMED

1. danshen[Title/Abstract]
2. compound salvia pellet[Title/Abstract]
3. danshen pill[Title/Abstract]
4. Danshen droplet pill [Title/Abstract]
5. or/1-4
6. "Angina, Stable"[Mesh]
7. angina[Title/Abstract]
8. or /6-7
9. 5 and 8

**EMBASE**

1. danshen. tw.
2. compound salvia pellet. tw.
3. danshen pill. tw.
4. Danshen droplet pill. tw
5. or/1-4
6. exp angina/
7. angina. tw.
8. or/6-7
9. 5 and 8

**Cochrane Central Register of Controlled Trials (CENTRAL)**

1. MeSH descriptor: [Angina, Stable] explode all trees
2. “angina”:ti,ab,kw
3. or/1-2
4. danshen:ti,ab,kw
5. compound salvia pellet:ti,ab,kw (Word variations have been searched)
6. “danshen pill”:ti,ab,kw
7. “danshen droplet pill” :ti,ab,kw
8. or/4-7
9. 3 and 8

CBM

1. 心肌梗塞[不加权:扩展]
2. 心梗[常用字段:智能]
3. 心肌梗塞[常用字段:智能]
4. Or/1-3
5. 丹参[常用字段:智能]
6. 4 and 5

CNKI

1. 心肌梗死[摘要]
2. 心梗[摘要]
3. Or/1-2
4. 丹参[摘要]
5. 3 and 4

Wangfang

1. 心肌梗死[主题]
2. 心梗[主题]
3. Or/1-2
4. 丹参[主题]
5. 3 and 4

Vip

1. 心肌梗死[主题]
2. 心梗[主题]
3. Or/1-2
4. 丹参[主题]
5. 3 and 4
